# The Use of Artificial Intelligence in Head and Neck Cancers: A Multidisciplinary Survey

**DOI:** 10.3390/jpm14040341

**Published:** 2024-03-25

**Authors:** Caterina Giannitto, Giorgia Carnicelli, Stefano Lusi, Angela Ammirabile, Elena Casiraghi, Armando De Virgilio, Andrea Alessandro Esposito, Davide Farina, Fabio Ferreli, Ciro Franzese, Gian Marco Frigerio, Antonio Lo Casto, Luca Malvezzi, Luigi Lorini, Ahmed E. Othman, Lorenzo Preda, Marta Scorsetti, Paolo Bossi, Giuseppe Mercante, Giuseppe Spriano, Luca Balzarini, Marco Francone

**Affiliations:** 1 Department of Biomedical Sciences, Humanitas University, Pieve Emanuele, 20072 Milan, Italygianmarco.frigerio@humanitas.it (G.M.F.); luigi.lorini@humanitas.it (L.L.); paolo.bossi@hunimed.eu (P.B.);; 2 Department of Diagnostic and Interventional Radiology, IRCCS Humanitas Research Hospital, Via Manzoni 56, 20089 Milan, Italy; 3 Department of Computer Science “Giovanni degli Antoni”, University of Milan, Via Celoria 18, 20133 Milan, Italy; elena.casiraghi@unimi.it; 4 Environmental Genomics and Systems Biology Division, Lawrence Berkeley National Laboratory, 717 Potter Street, Berkeley, CA 94710, USA; 5 Otorhinolaryngology Unit, IRCCS Humanitas Research Hospital, Via Manzoni 56, 20089 Milan, Italy; 6 Department of Diagnostic Radiology, ASST Bergamo Ovest, 24047 Treviglio, Italy; rxandreaesposito@yahoo.it; 7 Department of Medical and Surgical Specialties, Radiological Sciences and Public Health, University of Brescia ASST Spedali Civili of Brescia, 25123 Brescia, Italy; davide.farina@unibs.it; 8 Department of Radiotherapy and Radiosurgery IRCCS Humanitas Research Hospital, Via Manzoni 56, 20089 Milan, Italy; 9 Department of Biomedicine, Neuroscience and Advanced Diagnostics (BIND), University Hospital of Palermo, 90127 Palermo, Italy; antonio.locasto@unipa.it; 10 Medical Oncology and Hematology Unit IRCCS Humanitas Research Hospital, Via Manzoni 56, 20089 Milan, Italy; 11 Department of Neuroradiology, University Medical Center Mainz, 55131 Mainz, Germany; ahmed.othman@unimedizin-mainz.de; 12 Radiology Unit, Department of Clinical, Surgical, Diagnostic and Pediatric Sciences, University of Pavia, 27100 Pavia, Italy; lorenzo.preda@unipv.it

**Keywords:** artificial intelligence, multidisciplinary care, head and neck cancers, survey, diagnostic accuracy

## Abstract

Artificial intelligence (AI) approaches have been introduced in various disciplines but remain rather unused in head and neck (H&N) cancers. This survey aimed to infer the current applications of and attitudes toward AI in the multidisciplinary care of H&N cancers. From November 2020 to June 2022, a web-based questionnaire examining the relationship between AI usage and professionals’ demographics and attitudes was delivered to different professionals involved in H&N cancers through social media and mailing lists. A total of 139 professionals completed the questionnaire. Only 49.7% of the respondents reported having experience with AI. The most frequent AI users were radiologists (66.2%). Significant predictors of AI use were primary specialty (V = 0.455; *p* < 0.001), academic qualification and age. AI’s potential was seen in the improvement of diagnostic accuracy (72%), surgical planning (64.7%), treatment selection (57.6%), risk assessment (50.4%) and the prediction of complications (45.3%). Among participants, 42.7% had significant concerns over AI use, with the most frequent being the ‘loss of control’ (27.6%) and ‘diagnostic errors’ (57.0%). This survey reveals limited engagement with AI in multidisciplinary H&N cancer care, highlighting the need for broader implementation and further studies to explore its acceptance and benefits.

## 1. Introduction

Head and neck (H&N) cancers rank as the seventh most common malignancies worldwide, with 1.1 million new cases annually [1,2], and their incidence is expected to increase by 30% by 2030 [3,4]. Only one-third of HNSCC patients are diagnosed at an early stage, when it can be managed with surgery or radiotherapy with a 70–90% cure rate. The remainder two-thirds of patients are diagnosed at advanced stages, such as the T3-T4 or metastatic stage, and require multimodal treatment [5,6]. The outcome is variable and largely dependent on patient characteristics and on disease-specific features; this is why a personalized approach is fundamental [7]. Integrating data from oncology, surgery, imaging and radiotherapy in a multidisciplinary team (MDT) setting is the most effective way to design patient-tailored strategies; it has shown to impact decision making and survival rates and reduce hospital-related costs [8,9,10,11,12,13]. Artificial intelligence constitutes an ideal tool for integrating such multimodal data from the MDT setting, which is why it has gained the interest of the scientific community in the past few years. One important example of AI application is machine learning, which is now increasingly utilized for combining genetic, clinical, serological and multi-omics data and generating prognostic and predictive algorithms. These formulas can accurately infer patient-specific outcomes and thus help make the most appropriate clinical decisions [9,14]. Clinical applications of AI in oncology include screening, risk stratification, shared decision making and the allocation of the best treatments to patients [8,9]. In the field of surgery, artificial intelligence (AI) is employed for the delineation of surgical anatomy, augmentation of visual and manual skills and preoperative evaluation [10]. Among all specialties involved in cancer care, radiology has witnessed the most extensive AI application, with imaging-related tools accounting for around 50% of all AI cancer-related applications [11]. AI and machine learning are used for lesion identification, image segmentation and reconstruction, radiomics analysis, reporting, study triage and the evaluation of treatment responses [12,13]. All of this imaging-derived information is fundamental for improving the quality of treatment and better estimating patient prognosis [8,14,15]. For all of the above-mentioned advantages, AI is gaining a key role in the multidisciplinary evaluation of many cancer types, especially breast, prostate and lung cancers [11,16]. However, H&N oncology has been slow to adopt AI tools, mainly due a lack of confidence and awareness of AI and its potential applications [17].

The purpose of this survey was to infer the general knowledge, awareness, beliefs and concerns regarding AI applied to the management of H&N tumors.

## 2. Materials and Methods

Approval by our institutional review board was waived before the start of the present prospective study. Data collection and processing were performed in conformity with principles stated in the Declaration of Helsinki [18].

A web-based, closed questionnaire consisting of 19 items was designed by a radiologist (C.G.), who is an expert in the field of H&N oncology and a member of the tumor board of our academic hospital. The survey was distributed via Google Forms (11 February 2020 to 3 June 2022), with access provided through a link or QR code; the target population was members of the H&N MDT, including ENT doctors, surgeons, radiologists, pathologists, endocrinologists and physicians, and all levels of medical education were addressed (PhD doctors, medical doctors, research fellows, interns and professors). The questionnaire was delivered through social media (LinkedIn, Facebook and Researchgate) from November 2020 to June 2022, and through the mailing lists of the members of the European Society of Head and Neck Radiology (ESHNR) and the Italian Society of Radiology (SIRM), who were invited to share the survey with the colleagues of their H&N MDT. It was also administered during H&N meetings and during the annual meeting of the Italian Association of Otolaryngology and Head and Neck Surgery (SIOeChCF). Wording was formulated to avoid influencing the participants, who were informed about the length of the survey, the purpose and the rationale of the study. Informed consent was waived before the questionnaire was completed. Participation was voluntary and anonymous, with the possibility of leaving as many questions blank as desired; neither time limits nor incentives were given for completion of the survey. Users were prevented from accessing the survey more than once, so duplicate entries were avoided. Results were reported following the Checklist for Reporting Results of Internet E-Surveys (CHERRIES) [19].

### Survey Content and Data Elaboration

Online access to the form and answers was restricted to the principal investigator (C.G.) and two members of the research group (S.L. and G.C.) who were in charge of data elaboration. Five sections were investigated: socio-demographic data (including the age and sex of participants and the country where they practiced), ‘professional profile’ (specialty, academic position and working environment), ‘use of AI in clinical practice’, ‘potential applications of AI’ and its ‘perceived relevance’. The complete list of survey questions and multiple-choice answers is reported in Table S1.

SPSS version 27 software was used for statistical analysis (IBM Corp., released in 2020, IBM SPSS Statistics for Windows, Version 27.0, Armonk, NY, USA: IBM Corp), which involved descriptive and inferential statistics. Fisher’s exact test was used to infer the association between the use of AI (dependent variable) and variables among specialty, academic profile, age, country and academic qualification; Cramer’s V was used for the estimation of the effect size. A threshold of *p* ≤ 0.05 was set for statistical significance.

## 3. Results

### 3.1. Sociodemographic Characteristics and Professional Profiles

**Figure 1:**
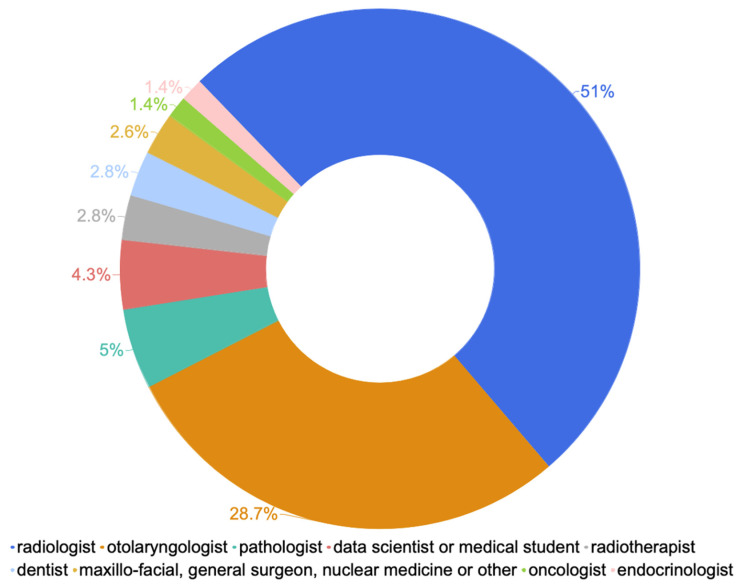
Distribution of medical specialties within population sample.

A total of 139 participants answered the survey, among which 71 (51%) were radiologists, 40 (28.7%) were otolaryngologists, 4 (2.8%) were radiotherapists, 2 (1.4%) were oncologists, 7 (5%) were pathologists and 4 (2.8%) were dentists. The other 4.3% comprised data scientists and students, and 1.4% comprised endocrinologists. Other less represented specialties were maxillo-facial specialists, general surgeons, and nuclear medicine specialists (2.6%) (Overall Answer Rate (AR) = 100%; Figure 1).

The age range was homogeneous: 13% of the participants were less than 30 years old, 31.5% were between 30 and 39, 27.3% were between 40 and 49, 16% were between 50 and 60 and 12% were greater than 60 (AR = 100%).

Most participants (70.3%) worked in teaching hospitals; only 18.2% were from non-academic hospitals, and 9.1% practiced privately (AR = 100%). 

**Figure 2:**
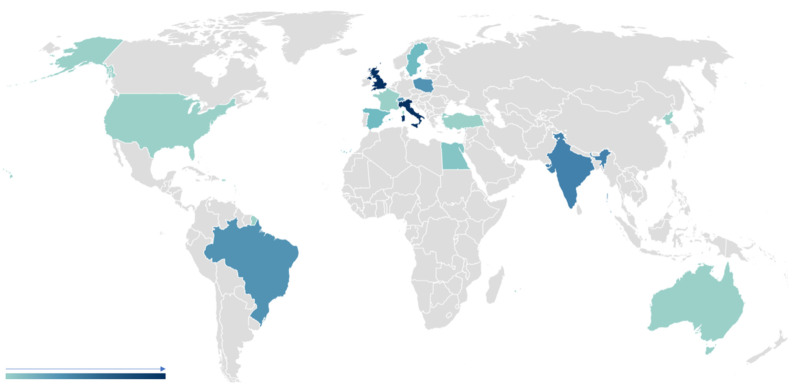
Geographic distribution of survey respondents.

The geographic distribution was heterogeneous with 14 countries represented: the majority of the respondents came from European countries (75%; 65% were from Italy, and the rest were from France, Spain, Poland and Sweden), 9.2% were from the United Kingdom and 1.6% were from Australia and the United States; the remainder were from India (4.2%), Brazil and Switzerland (3.3% each), Egypt (1.6%) and Republic of Korea and Turkey (0.8% each; AR = 83%; Figure 2).

### 3.2. Knowledge and Factors Associated with Use of Artificial Intelligence

Only half of the study population reported having had experience with AI (49.7% users versus 50.3% non-users; AR = 100%).

**Figure 3:**
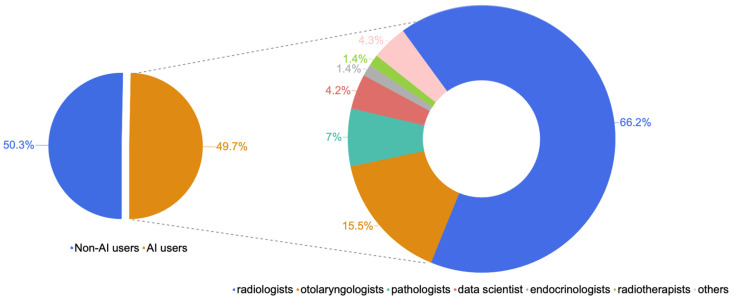
Distribution of specialties within AI users.

Radiologists accounted for 66.2% of AI users, otolaryngologists accounted for 15.5%, pathologists accounted for 7%, data scientists accounted for 4.2% and endocrinologists and radiotherapists accounted for 1.4% each; none of the oncologists interviewed reported ever using AI (Figure 3). Regarding the frequency of AI use within specialty (intra-class evaluation), the highest prevalence was among pathologists (71.4% prevalence within the specialty), followed by radiologists (66.4%). AI use was less frequent among otolaryngologists (ENT specialists, 26%) and radiotherapists (25%).

**Figure 4:**
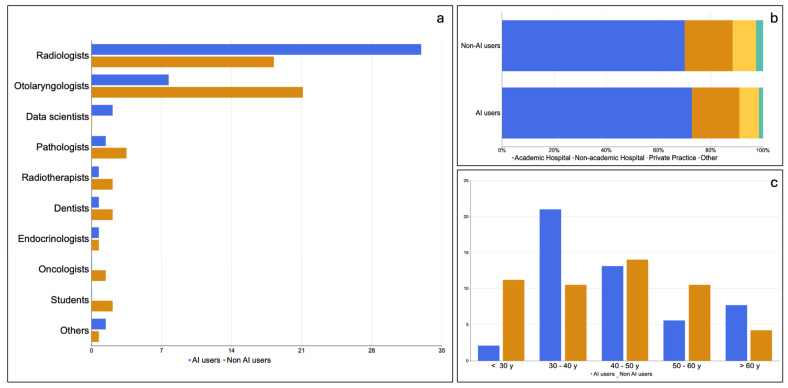
Prevalence of artificial intelligence use (**a**) within each specialty, (**b**) within different working environments and (**c**) within age groups.

Most AI users (67.6%) worked in academic hospitals, with consultants and attendings representing the most frequent category (49.3% of AI users: 29.6% consultants and 19.7% attendings), followed by academic physicians (21%), medical residents (12.7%) and medical interns (2.8%); 5,2% were clinical fellows. The remainder 9% did not apply to the formerly mentioned categories and were classified as ‘other’ (hospital staff: nurses, case managers, psychologists in MDTs and private practitioners). The age of AI users was between 20 and 30 years in 42.3% of cases, between 30 and 40 in 26.8% of cases, above 60 in 15% of cases and in the 40–50 range in 11.3% of cases. The age range with the highest intraclass rate of AI use was between 30 and 40 (Figure 4).

The nonparametric Fisher’s exact test revealed a statistically significant association between the use of AI and primary specialty (V = 0.455; *p* < 0.001), with radiology having the strongest association (*p* < 0.001). Other significant predictors of AI use were academic qualification (V = 0.354; *p* < 0.05) and age (V = 0.35; *p* < 0.001); this was particularly true for MDT members between 30 and 40 years (*p* < 0.007) and less than 30 years of age (*p* < 0.01). Professionals involved in academic and scientific work were more likely to use AI (*p* = 0.05). Other relevant correlations, although without a statistical significance (*p* < 0.075), were geographic distribution, with Western European countries having, on average, higher knowledge and use of AI. No differences across genders (*p* = 0.86) or working environments (*p* = 0.5) were reported. Among all of the respondents, 8% did not know the meaning of AI (AR = 100%). The participants were asked to rate the importance of AI in their practice; AI use was considered essential by 4.4% of the study participants and important by 84%; 3% of respondents rated it not important, and 8.6% were not able to express an opinion about AI usage in H&N oncology (AR = 90%).

### 3.3. Applications, Concerns and Perceived Importance of Artificial Intelligence

The most frequent application of AI was to the interpretation and quantification of imaging findings (70.8% of respondents), followed by computer-aided diagnosis (45.8%), grading disease severity (37.5%), outcome prediction (32%), patient information and shared decision making (18.1%) and the prediction of complications (14%). Other minor applications (7%) reported were pre-operative surgical planning and research purposes.

Diagnostic imaging was the specialty where AI was applied most frequently (84% answers), followed by radiation oncology (72%), oncology (67.2%), surgery (42%) and research (52%) (AR = 90%).

When asked for the most relevant benefits resulting from AI use, the majority of the study participants reported improved accuracy of diagnosis (72%), followed by advantages in surgical planning (64.7%) and treatment selection (57.6%). Other reported benefits were the reduction in time associated with medical procedures (52%), risk assessment (50.4%), the prediction of complications (45.3%), shared decision making (39%) and screening (0.7%; AR = 89%).

**Figure 5:**
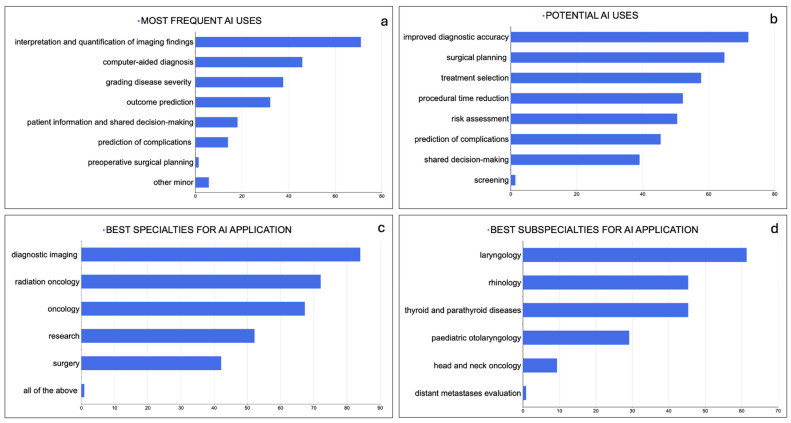
Perceived value of AI (**a**) in medical specialties, (**b**) in potential subspecialty areas, (**c**) in current common uses and (**d**) in predicted future beneficial applications.

The participants were asked to list sub-fields of ENT practice in which AI would provide significant improvements (potential applications); skull base surgery was the most frequently reported field (66.9%), followed by laryngology (61.3%), rhinology (45.2%), thyroid and parathyroid diseases (45.2%) and pediatric otolaryngology (29%); 6% of respondents would consider AI application for the treatment of other H&N cancers (Figure 5). Individual responses to open questions on the specific applications of AI related to ENT pathology are listed in Table S2 (AR = 37%).

**Figure 6:**
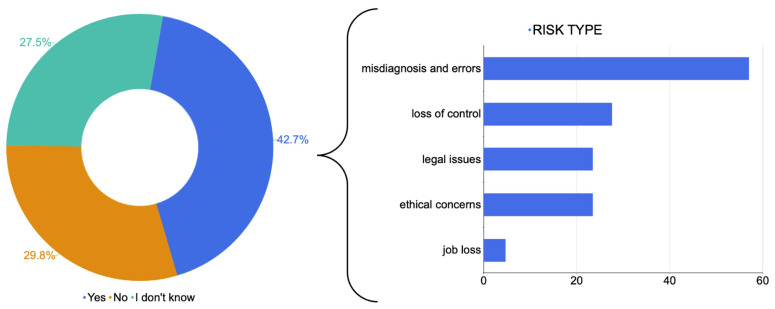
Attitudes of participants towards artificial intelligence and categories of risks associated with artificial intelligence.

When asked whether risks were foreseen related to the use of AI, the majority of the responders (42.7%) had significant concerns, 29.8% had no concerns at all and the remaining 27.4% did not take a position (AR = 89%). The participants were asked to express their main concerns by answering an open question (answers are reported in Table S3): the answers were summarized in five major groups: ‘loss of control’ (27.6%), ‘legal issues’ (23.4%), ‘ethical concerns’ (23.4%), ‘misdiagnosis and errors’ (57%) and ‘job losses’ (4.7%; AR = 37%; Figure 6).

## 4. Discussion

AI has witnessed exponential growth in recent years, with numerous surveys assessing the knowledge and perception of it in clinical practice. Even so, no studies focused on the perception of AI in H&N oncology have been performed even though this tool has the potential to dramatically improve H&N care. In fact, the skills and knowledge required for its use remain largely unrecognized by H&N healthcare professionals [17,20].

Our multidisciplinary survey collected data from a heterogeneous cohort of physicians involved in H&N cancers across different levels of training. Despite the wide popularity gained in the past decade, less than half (49.7%) of the participants had ever experienced AI, and 8% of them did not even know the meaning of the term. Among AI users, some specialties were significantly better represented; radiologists accounted for 51% of all users, with an intraclass prevalence of 66.45%. A high prevalence of AI use was also found among pathologists (71%). These findings are consistent with recently published studies in the literature [21,22] that reflect the suitability of AI tools for pattern recognition and feature classification, which are characteristics that relate most to diagnostic specialties [11]. According to correlation analysis, significant predictors of AI use were primary specialty (V = 0.455; *p* < 0.001), with radiology having the strongest association (*p* < 0.001), followed by young age (*p* < 0.007) and academic qualification. 

Users were more likely to work in teaching hospitals and were more often faculty members, PhD students or residents. On the other hand, AI use was relatively rare among private practitioners, attendings and non-academic physicians. This could suggest that AI remains a prerogative of the academic community and of younger generations that are more acquainted with digital technology. Among the 14 countries included, Northern European and Western countries had the highest prevalence of AI use, which may indicate a higher tendency for high-income countries to employ AI tools [21,23,24,25]. 

The awareness of AI and its potential applications still remains scarce, especially among ENT specialists (prevalence of AI use of only 26%) and surgeons, despite them being critical figures in the primary care of H&N cancers.

In line with the data from the current literature [24], the most frequently reported uses of AI were related to imaging, particularly the interpretation and quantification of imaging findings (70.8% of respondents), texture analysis and computer-aided diagnosis (45.8%), and the grading of disease severity (37.5%). Likewise, when asked which area would most benefit most from AI implementation, most study participants would recommend AI for improving the accuracy of diagnoses (72%).

Other frequent applications were clinical; in particular, AI was often employed for predicting outcomes and complications (32% and 14%, respectively). Patient information and shared decision making (18.1%) were also reported as common areas of AI use, suggesting that AI may significantly improve patient–doctor communication [26]. Previous studies have extensively validated the use of AI in improving decision making [27]; in a cohort study enrolling over 33,000 patients, Howard et al. evaluated the performance of three machine learning models in identifying H&N cancer patients that could benefit from adjuvant chemotherapy, with a survival benefit of up to 80% (HR 0.83; *p*  <  0.001) compared to the standard of care [28]. AI has also been applied in the assessment of important clinical parameters, like human papillomavirus (HPV) status (reported prediction accuracy from 70 to 80%) [29,30,31,32], for the planning of radiotherapy [33,34,35] and the prediction of toxicity [36]. The use of AI in H&N network medicine has already demonstrated promising results, as exemplified by the “The Big Data to Decide (BD2Decide)” project, i.e., the largest database built on HNSCC. Multi-omics data collected from a dataset of 1537 stage III-IV HNSCC cases have been used to develop AI-based prediction models, ranging from prognosis estimation to outcome prediction and the allocation of treatments to patients [37].

Surprisingly, over 60% of respondents suggested surgery as a potential field of future application, namely surgical pre-planning, reducing procedural times (52%) and surgical subspecialties such as skull base surgery (66.9%). Several works have already been published on AI use for surgical decision making, the prediction of surgical outcomes and the establishment of surgical approaches [22,38,39,40], further endorsing the value of AI in this field.

Our survey revealed interesting insights into the fears and concerns associated with AI use, which we summarized into five main categories: ‘loss of control’, ‘legal issues’, ‘ethical concerns’, ‘diagnostic errors’ and ‘job losses’. Almost half of the study population (42%) foresaw significant risks related to AI employment. The most frequently reported was the occurrence of errors and misdiagnosis (57%).

AI algorithms are liable to errors despite being designed with the aim of minimizing human mistakes. In a landmark paper by Amodei et al. [41], five error types or ‘accidents’ are described as being related to discrepancies between training datasets and real-world data, the misinterpretation of data and wrong associations. Furthermore, AI algorithms may fail to consider the wide-context picture or may inadvertently produce unsafe conditions to test hypotheses [41,42]. The same issue can be related to the currently popular chatbox, Chat GPT-4; in a critical appraisal of this newly released AI tool, Lee et al. [43] described errors or so-called ‘hallucinations’ produced from either the misinterpretation of the ‘prompt’ (the main question of the session) or the formulation of inappropriate solutions. A further concern associated with AI use was the loss of medical knowledge, which may one day hamper progress and autonomy [42,44]. The decline in medical proficiency also implies a loss of control over AI, a concern mentioned by 27.6% of our study participants. Other significant apprehensions regarding AI in medical practice were in the ethical and legal fields (23% of reported concerns). The main legal issues regard accountability for medical actions. When the caregiver is no longer in control of decision making, they cannot be considered responsible for any harm to the patient [45]. A second major issue concerns the ethics of medical practice, according to which the patient–doctor relationship is a cornerstone. AI utilization may dramatically impact the moral framework of our society in addition to generating problems of confidentiality and data sharing [46]. The same fears are also reported by patients; as a survey from Richardson et al. showed, patients tend to be concerned about medical errors and the loss of contact with doctors, and they fear the inability to choose their treatment among a variety of options. The effect of such concerns is that most patients would avoid the use of AI [47].

To minimize the risks related to accountability and safety, regulatory guidelines are being released on AI use and methodology. One example is the U.S. Food and Drug Administration. The ‘Clinical Decision Support Software–Guidance for Industry and Food and Drug Administration Staff’ (2022) is a regulatory framework for AI software use provided by the FDA (available at https://www.fda.gov/regulatory-information/search-fda-guidance-documents/clinical-decision-support-software, accessed on 19 February 2024). These regulations highlight the need for supervision on AI and warn about the risks of automation bias [48]. AI should be regarded as a precious tool to augment human capabilities; however, it should guide medical decisions rather than replace them [42].

This study is significant for two main reasons. First, it offers an explorative summary of the subjective knowledge and attitudes towards AI. Second, it outlines the potential issues that might arise during the development and implementation process of AI-based technologies. The qualitative nature of this study assesses the factors influencing subjective attitudes and perspectives toward specific benefits or challenges associated with AI development, use and implementation. However, participants’ expectations may be subjectively influenced by their own personal experience with AI systems. Future research should validate the results of this study to establish a reliable association between the perception and acceptance of AI.

We are aware of some limitations of the current study. Its explorative and descriptive findings might not be generalizable to the wider H&N community. Furthermore, the limited sample size and the unbalanced composition of the population subgroups could be related to the selective distribution of the questionnaire within specific scientific societies, mainly among members of radiology and ENT societies. The nature of the sampling method might have introduced some biases, particularly concerning interest in the topic or willingness to participate. In addition, no preventive testing to assess usability and technical functionality was performed prior to administering the questionnaire to the target population. To improve these limitations and to collect follow-up data, future studies should employ a more diverse sampling strategy to achieve a better balance of individual interests in the topic. We plan to formulate a new version of the same survey, expanding our reach to global H&N societies through an “AI-awareness initiative”.

## 5. Conclusions

Our study is the first to our knowledge to provide an overview on the perception, attitudes and trust toward AI among H&N healthcare professionals. This study has shown a lack of knowledge about AI and how it can improve daily clinical practice; hence, collecting data on its use and on the opinions around it is of paramount importance. Awareness should be raised in the future over AI and its related tools, through sensitization programs, to help H&N-MDT clinicians embrace progress and avoid skepticism. Our survey sheds light on which fears still need to be overcome, especially in regard to diagnostic accuracy and ethical issues. If used with cautious and constant clinical supervision, AI can be a game changer in the management of cancer patients.

## Data Availability

The data that support the findings of this study are available from the corresponding author, A.A., upon reasonable request.

## Supplementary Materials

The following supporting information can be downloaded at https://www.mdpi.com/article/10.3390/jpm14040341/s1, Table S1: Original survey questions and relative response options. Table S2: A list of the most frequent responses to the following question: *“Is there any particular procedure which would benefit from AI implementation?”* Table S3: A list of the most frequent responses to the following question: *“Which risks are you foreseeing in regard to AI use?”*.
