# Supplementary material for: The Use of Artificial Intelligence in Head and Neck Cancers: A Multidisciplinary Survey
Source: J Pers Med. 2024 Mar 25;14(4):341. doi: 10.3390/jpm14040341 (PMC11050769; doi:10.3390/jpm14040341)
Supplement: Supplementary file 1 [file jpm-14-00341-s001.zip › jpm-2901573-supplementary.pdf]

Supplementary Material

Use of artificial intelligence in Head and Neck cancers: a multidisciplinary survey

Table S1. Original survey questions and relative response options.

Domain	Question	Question type	Listed options *
Socio-demographic characteristics	What is your gender?	Multiple choice	Male, Female, Prefer not to say
	What age group do you belong to?	Multiple choice	<30 years, 40–49 years, 50–60 years, >60 years
	What country are you currently based in?	Multiple choice	List of countries*
Professional profile	What is your primary specialty?	Multiple choice	Otolaryngologist, Maxillo-facial surgeon, General surgeon, Pathologist, Radiologist, Endocrinologist, Radiation oncologist, Other (please specify)
	What is your affiliation (Hospital)?	Open question	\
Use of AI in clinical practice	What is your affiliation (Department)?	Open question	\
	What setting do you primarily practice in?	Multiple choice	Academic hospital, Non-academic hospital, Public/private hospital or clinic, Other (please specify), …
	What is your level of experience?	Multiple choice	Resident, Fellow, PhD, Attending, Consultant, Student, Other (please specify)
	How would you define AI?	Multiple choice	Simulation of human behaviour by computerized systems, Building memory in a computer, I don't know, ….
Potential applications of AI	What are the key technologies behind AI?	Multiple choice	Machine learning, Robotics, Electronic, Blockchain, I don't know, ….

	Have you ever used AI techniques/applications in your clinical practice?	Multiple choice	Yes, No, Other (please specify)

Abbreviations: AI = Artificial Intelligence

Table S2. A list of the most frequent responses to the following question *"Is there any particular procedure which would benefit from AI implementation?"*

Is there any particular procedure which would benefit from AI implementation?
Identification of surgery-related major risks and ways to reduce them (e.g. identification of anatomical variations that could increase the risk of the intervention)
AI could be helpful in selecting the most appropriate therapy considering the risks of surgery and the survival rates with or without radio- and chemotherapy
In the field of radiotherapy, AI could enhance the definition of irradiated fields
Computer-assisted diagnosis
Systematic depiction of dysphagia lusoria by using US/Chest CT before right lobe thyroid surgery
Preoperative assessment of skull base tumors
Minimally invasive ultrasound guided RFA
Prototyping for facial bone reconstruction
CT, MRI, PET imaging, Conventional radiology
Imaging follow-up after therapy. Prognostic information or therapeutic option selection based on large datasets of both imaging information, surgical complication, additional treatments, (epi)genetic factors and epidemiological information
Imaging acquisition, texture-based image interpretation and decision making, especially treatment selection
Robotic surgery, videoscopy, image-Enhanced Endoscopy
Enhancing imaging workflows, improving efficiency of protocolling and quality of images, and aiding with diagnosis and quantitative analysis
Scoring thyroid nodules on ultrasound. Scoring severity of sinus mucosal pathology. Calculating risks and benefits of treatment on an individual basis
Anatomical segmentation in radiology and radiotherapy, anatomical delineation for pre-surgical planning (e.g. FESS), thyroid nodule risk stratification, parathyroid detection
Optimization of dose and therapy fields in radiation oncology
Post-radiation thyroid imaging
Risk stratification
Triage for urgent imaging and treatment. Patient assessment for risk of recurrence. Imaging: detection of lymph node metastases, Sjogren Syndrome, thyroid nodule classification (TI-RADS) and decision making on FNA, bone invasion by cancer, post- radiotherapy changes vs recurrence

Ultrasonography for thyroid nodules
Image-guided biopsy
Early cancer screening
Early detection of microlesions and micrometastases
Standardization of imaging analysis

Abbreviations: AI = Artificial Intelligence; CT = Computed Tomography; FESS = Functional Endoscopic Sinus Surgery; FNA = Fine Needle Aspiration; MRI = Magnetic Resonance Imaging; PET = Positron Emission Tomography; RFA = Radiofrequency Ablation; TI-RADS = Thyroid Imaging Reporting and Data System; US = Ultrasound.

Table S3. A list of the most frequent responses to the following question *"Which risks are you foreseeing in regard to AI use?"*

Which risks are you foreseeing in regard to AI use?
Unpredictability
Dependence upon technology, hampering critical thinking
Excessive trust in AI, leading to overlooked clinical judgment
Anatomical variants that may not be recognized by the AI
Necessity to clarify that final decisions should always rely on the physician or surgeon according to his knowledge and understand about AI use (loss of control)
Loss of autonomy, laziness of clinicians and users; excessive dependency from technology; loss of control over the decision-making process; loss of critical thinking and clinical reasoning
Risk of relying on AI without using natural intelligence
Potential issues related to data protection
Complications
Overfitting of models and lack of reproducibility
Loss of patient-tailored approach, due to therapies and decisions made solely based on statistical evidence
Underdiagnosis; overlooked diagnoses; Diagnostic errors
Absence of homogeneous standardized results
Excessive advanced AI algorithms that prevented a full control on the machine outcomes, forcing to have faith or just to ignore the results
Errors in calculating machine learning outcomes
Need of AI to integrate clinical and pathologic data in order to ensure a comprehensive approach
Observer Bias
Excessive use in clinical setting as a cause of a loss of empathy for patients, a core value in patient care
Use of AI without proper training
Poor data input as a cause of low-quality algorithms, not applicable to the general population
Legal issues
Ethical concerns; low quality data and/or analysis
Job loss, particularly in scenarios where AI handles standard cases, leaving complex cases to clinicians

Accountability for medical actions and ethics and compliance; Legal issues for missed or misinterpreted results
Ethics; liability law; misdiagnosis with missing critical review....
Inequality of care (due to access to technology, cost)
Data privacy of patients, bias of algorithms
Accountability and legal framework for mistakes made by AI/machine learning algorithm
Delegating decisions to AI
Risk of misinterpretation in surgery for complete AI interpretation of imaging; need of a huge amount of data for a fair dataset training
Human and automation bias
Misinterpretation
The loss of human supervision and review
Misclassification of a lesion by the algorithm; need of human final decision
Understanding how the model works. Blackbox model phenomenon. Poor quality of AI algorithm. Limited number of cases used for machine learning
Legal issues for the responsibility of errors among doctors, institution which implemented the system or developing company. Need of constant update of the algorithms developed by engineers for the risk of misleading output.
Need of a regulatory framework oversighted by clinicians, to avoid false reassurance falsely and increased rate of unnecessary imaging.
Too strong relying on AI and basing decisions only on its outputs
Governance of both product generation and sustenance
Dependance of AI outcome from the training dataset. Risk of "Garbage in- garbage out" phenomenon affecting performance
Bad usage of data, data leakage
Excessive influence by AI tools on less skilled user
Excessive trust on AI results by radiologists, overlooking diagnostic examinations

Abbreviations: AI = Artificial Intelligence
